# TFAP2C increases cell proliferation by downregulating GADD45B and PMAIP1 in non-small cell lung cancer cells

**DOI:** 10.1186/s40659-019-0244-5

**Published:** 2019-07-11

**Authors:** Hyunhee Do, Dain Kim, JiHoon Kang, Beomseok Son, Danbi Seo, HyeSook Youn, BuHyun Youn, Wanyeon Kim

**Affiliations:** 1 Department of Science Education, Korea National University of Education, Cheongju-si, Chungbuk 28173 Republic of Korea; 2 Department of Integrated Biological Science, Pusan National University, Busan, 46241 Republic of Korea; 3 Department of Integrative Bioscience and Biotechnology, Sejong University, Seoul, 05006 Republic of Korea; 4 Department of Biological Sciences, Pusan National University, Busandaehak-ro 63beon-gil, Geumjeong-gu, Busan, 46241 Republic of Korea; 5 Department of Biology Education, Korea National University of Education, 250 Taeseongtabyeon-ro, Gangnae-myeon, Heungdeok-gu, Cheongju-si, Chungbuk 28173 Republic of Korea

**Keywords:** TFAP2C, Tumor suppressor, GADD45B, PMAIP1, Lung tumorigenesis

## Abstract

**Background:**

Non-small cell lung cancer (NSCLC) is one of the leading causes of death in the world. NSCLC diagnosed at an early stage can be highly curable with a positive prognosis, but biomarker limitations make it difficult to diagnose lung cancer at an early stage. To identify biomarkers for lung cancer development, we previously focused on the oncogenic roles of transcription factor TFAP2C in lung cancers and revealed the molecular mechanism of several oncogenes in lung tumorigenesis based on TFAP2C-related microarray analysis.

**Results:**

In this study, we analyzed microarray data to identify tumor suppressor genes and nine genes downregulated by TFAP2C were screened. Among the nine genes, we focused on growth arrest and DNA-damage-inducible beta (GADD45B) and phorbol-12-myristate-13-acetate-induced protein 1 (PMAIP1) as representative TFAP2C-regulated tumor suppressor genes. It was observed that overexpressed TFAP2C resulted in inhibition of GADD45B and PMAIP1 expressions at both the mRNA and protein levels in NSCLC cells. In addition, downregulation of GADD45B and PMAIP1 by TFAP2C promoted cell proliferation and cell motility, which are closely associated with NSCLC tumorigenesis.

**Conclusion:**

This study indicates that GADD45B and PMAIP1 could be promising tumor suppressors for NSCLC and might be useful as prognostic markers for use in NSCLC therapy.

**Electronic supplementary material:**

The online version of this article (10.1186/s40659-019-0244-5) contains supplementary material, which is available to authorized users.

## Background

Lung cancer is one of the most common cancers and is the leading cause of cancer-related deaths worldwide. Lung cancer is divided into small cell lung cancer and non-small cell lung cancer (NSCLC) with NSCLC responsible for almost 80% of lung cancer-related deaths [1, 2]. Despite improvements in chemotherapy and molecular-targeted therapy over recent decades, the 5-year survival rate of NSCLC patients is only 14% [2, 3]. Patients with NSCLC can have a greatly improved prognosis and survival rate when they are diagnosed at an early stage. However, early diagnosis of NSCLC is difficult due to biomarker limitations. Discovery of tumor-specific factors that can influence the diagnosis of NSCLC is critical. Thus, a study to identify novel molecular targets of NSCLC can contribute to improvements in the early diagnosis and therapy of NSCLC.

Transcription factor activating enhancer-binding protein 2C (TFAP2C) is a member of the AP-2 family which has five isoforms. TFAP2C was first shown to play a role in embryonic development [4]. Subsequently, accumulated data have provided several clues that TFAP2C is also highly involved in human solid cancers. TFAP2C is responsible for angiogenesis via an increase in the expression of the extracellular matrix 1 gene in melanoma [5]. In luminal breast cancer, TFAP2C increases epidermal growth factor receptor expression, resulting in cancer cell proliferation and tumor growth [6]. To elucidate a role of TFAP2C in NSCLC, we previously reported that TFAP2C mediates inhibition of A-kinase anchor protein 12 and activation of cell division protein kinase 6 through upregulation of miR-183 and downregulation of miR-33a, leading to cell cycle progression and NSCLC malignancy [7]. TFAP2C can also increase the expression level of transforming growth factor beta receptor I, consequently promoting NSCLC cell proliferation [8]. Recently, it was reported that TFAP2C participates in the enhancement of cancer stemness and chemoresistance in colorectal cancer [9]. These studies have shown that TFAP2C is significantly involved in various cancers; an involvement that may depend on environmental conditions and tumor type. Although these previous studies have indicated the importance of TFAP2C in cancer progression, its roles in NSCLC remain to be investigated.

The present study was established to identify novel tumor suppressive genes that are downregulated during TFAP2C-mediated NSCLC tumorigenesis. Through an analysis of a TFAP2C-related microarray dataset, we identified nine candidate genes functioning as tumor suppressors. In particular, growth arrest and DNA-damage-inducible beta (GADD45B) and phorbol-12-myristate-13-acetate-induced protein 1 (PMAIP1) were downregulated by TFAP2C-overexpressing NSCLC cells, which contributed to NSCLC cell proliferation and motility. Our analysis has identified putative target genes for TFAP2C-mediated NSCLC tumorigenesis, and the results suggest that those genes might be used to provide prognostic targets in the treatment of NSCLC.

## Methods

### Antibodies and reagents

Antibodies specific for TFAP2C (sc-12762), tubulin (sc-23948), GADD45B (sc-377311) and PMAIP1 (sc-515840) were purchased from Santa Cruz Biotechnology (Santa Cruz, CA, USA). Cell culture media (DMEM and RPMI-1640), fetal bovine serum (FBS), penicillin and streptomycin were acquired from Gibco (Grand Island, NY, USA). Small interfering RNA (siRNA) specific for TFAP2C (sc-29696), GADD45B (sc-37416), and PMAIP1 (sc-37305) with control siRNA (sc-37007) were purchased from Santa Cruz Biotechnology.

### Cell lines and cell culture

The human normal lung cell line, WI-26 VA4, and four human NSCLC cell lines, A549, NCI-H292, NCI-H358, and NCI-H460, were acquired from the American Type Culture Collection (ATCC, Manassas, VA), authenticated, and then maintained in early passages for no more than 6 months after receipt from ATCC. The WI-26 VA4, A549, NCI-H460, NCI-H292, and NCI-H358 cells were grown in DMEM or RPMI-1640 supplemented with 10% FBS, 100 U/mL penicillin and 100 μg/mL streptomycin at 37 °C in a 5% CO_2_/95% air atmosphere.

### Transient transfection and real-time reverse transcriptase polymerase chain reaction (qRT-PCR)

A plasmid containing a full-length TFAP2C construct (pcDNA3.1-TFAP2C) and a control plasmid (pcDNA3.1 empty vector) were kindly provided by Ronald J. Weigel (University of Iowa, Iowa City, IA, USA). Cells were plated in 60 mm dishes before undergoing transfection. The cells were transiently transfected with pcDNA3.1-TFAP2C by using Lipofectamine RNAiMAX (Invitrogen, Carlsbad, CA, USA) and with si-TFAP2C, si-GADD45B, and/or si-PMAIP1 by using Lipofectamine 3000 (Invitrogen). After 48 h of transfection, the cells were collected and used for in vitro functional analyses. To analyze the expression levels of mRNAs [10], total RNA was isolated from cells using TRIzol (Invitrogen). To obtain cDNA, the isolated RNA was converted by using an MMLV Reverse Transcriptase system (Bioneer, Daejeon, Republic of Korea) according to the manufacturer’s protocol. The cycle parameters for the reverse transcription (RT) reaction were 25 °C for 5 min, 37 °C for 60 min, 95 °C for 5 min, and then held at 4 °C until used. Each RT product was used as a template for real-time quantitative PCR (qRT-PCR) with specific primers (Table 1). Aliquots of a master mix containing all reaction components and the primers were dispensed into a real time PCR plate (Applied Biosystems, Foster City, CA, USA). All PCR reagents were obtained from a SYBR Green core reagent kit (Applied Biosystems). Gene mRNA levels were measured in triplicate in the reaction plate. Real-time qRT-PCR was performed using an Applied Biosystems-7900 HT qRT-PCR instrument. PCR was performed over 40 cycles of 15 s at 95 °C and 1 min at 60 °C, after which samples were subjected to thermal denaturation. Gene expression levels versus *ACTN* mRNA were determined using the 2^−ΔΔCT^ method [11]. To simplify data presentation, relative expression values were multiplied by 10^2^.

**Table 1:** Primers for determining expression levels of TFAP2C, GADD45B and PMAIP1

Gene	Forward primer	Reverse primer
*TFAP2C*	5′-ACAGGATCCATGTTGTGGAAAATAACCGAT-3′	5′-ATACTCGAGTTTCCTGTGTTTCTCCATTTT-3′
*GADD45B*	5′-GTCGGCCAAGTTGATGAAT-3′	5′-CACGATGTTGATGTCGTTGT-3′
*PMAIP1*	5′-AGATGCCTGGGAAGAAG-3′	5′-AGTCCCCTCATGCAAGT-3′
*ACTN*	5′-TGAGAGGGAAATCGTGCGTG-3′	5′-TGCTTGCTGATCCACATCTGC-3′

### Western blot analysis

After experimental treatments, Western blotting was performed as previously described [12]. Whole-cell lysates and tissue lysates were obtained by using an EzRIPA Lysis kit (ATTO, Tokyo, Japan; 20 mM HEPES, pH 7.5, 150 mM NaCl, 1% NP-40, 0.1% SDS, 0.5% deoxycholic acid, as well as protease inhibitor and phosphatase inhibitor cocktails). Protein concentrations in lysates were determined by using a BioRad protein assay kit (BioRad Laboratories, Hercules, CA, USA). SDS-PAGE was performed with the protein samples, after which the proteins were transferred to a nitrocellulose membrane. For blocking, 5% bovine serum albumin in TBST (10 mM Tris, 100 mM NaCl, and 0.1% Tween 20) was used for 1 h at room temperature. Membranes were then incubated at 4 °C overnight with specific primary antibodies and subsequently probed with peroxidase-conjugated secondary antibodies (Santa Cruz) for 1 h at room temperature. Blots were visualized by using an ECL detection system (Abfrontier, Seoul, Republic of Korea). Data acquisition and densitometric analysis were performed using an iBright CL1000 imaging system (Thermo Fisher Scientific, Waltham, MA, USA).

### Cell viability assay

For cell viability assays, cells were cultured in 35-mm dishes at a density of 5 × 10^4^ cells. After 24 h, they were treated with TFAP2C overexpression and/or siRNA transfection. Cells were detached with 10% trypsin–EDTA and washed with PBS at the indicated times. The cells were resuspended in PBS and diluted 1:1 in trypan blue solution (Gibco). Cell viability was measured by determining the number of viable cells that excluded trypan blue solution.

### Colony-forming assay

For colony-forming assays [13], cells were plated at a density of 1000 cells per 35 mm dish. After 24 h, the cells were treated with the indicated gene and/or siRNA and then incubated at 37 °C in a 5% CO_2_/95% air atmosphere for 14 days. Subsequently, cells were fixed with 10% methanol, 10% acetic acid and stained with 1% crystal violet (Sigma, St. Louis, MO, USA). Colonies containing more than 50 cells were identified by using ImageJ software [14] and scored as survivors.

### Apoptosis assay

A Caspase-Glo 3/7 assay kit (Promega, Madison, WI) was used to detect apoptosis [15]. Briefly, treated cells (10^4^ cell per mL) in 100 μL of culture medium were transferred to a 96-well microplate, 100 μL of Caspase-Glo 3/7 reagent, which contained caspase 3/7 substrates, was added to each well. Well contents were gently mixed at 300 to 500 rpm for 30 s, and incubated at room temperature for 1 h. Sample luminescence was measured using a Glomax multidetection system (Promega).

### Wound-healing assay

For wound-healing assays [16], cells were cultured to 70% confluency in DMEM or RPMI-1640 medium with 1% FBS. The cell monolayers were then scratched with a 200 μL pipette tip. The cells were further incubated with fresh medium with or without treatment for 24 h or 48 h. Photomicrographs were taken at × 100 magnification with an Olympus CKX53 inverted microscope (Olympus Optical, Tokyo, Japan).

### Transwell cell migration assay

For cell migration assays [16, 17], cells (1 × 10^4^ in serum-free DMEM or RPMI-1640 medium) subjected to the desired treatments for 72 h were seeded into the upper chambers of a 24-well Transwell chamber (Corning, Corning, NY) fitted with a 5 μm pore-size insert. The lower chamber was filled with DMEM or RPMI-1640 medium containing 2% FBS. After 6 h, the upper membrane surface was wiped with a cotton swab to remove cells that had not migrated into the lower chamber. Cells that had migrated to the lower membrane surface were fixed with 4% paraformaldehyde, stained with hematoxylin, and counted. Migration indices were calculated and normalized to the number of control cells that had migrated. Results are expressed as fold increases in migration compared to control groups as determined by relative numbers of cells in a randomly selected field in experiments conducted in triplicate.

### Statistical analysis

All numeric data are presented as mean ± standard deviation (SD) values from at least three independent experiments and sample sizes were calculated to allow significance to be determined. Experimental results were analyzed by one-way ANOVA for ranked data followed by Tukey’s honestly significant difference test and by two-way ANOVA for ranked data followed by Bonferroni’s post-test. Prism 5 software (GraphPad Software, San Diego, CA, USA) was used to conduct all statistical analyses, and statistical significance was accepted for a *p* value < 0.05.

## Results

### Screening candidates for tumor suppressor genes regulated by TFAP2C in lung tumorigenesis

**Fig. 1:**
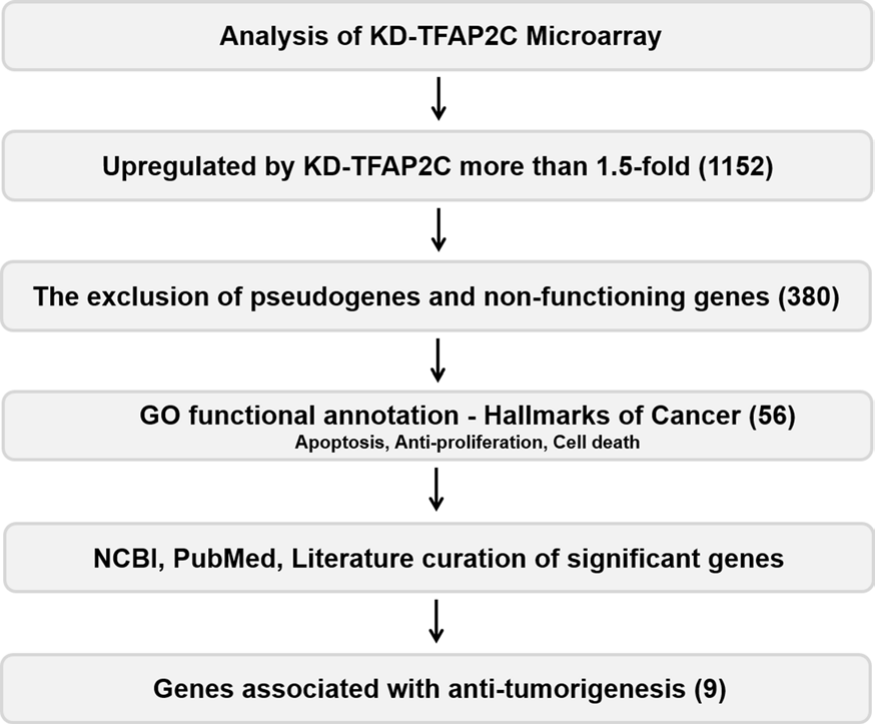
Schematic of the process used to identify TFAP2C-target genes functioning as tumor suppressors based on the microarray data (GSE79228)

Based on the role of TFAP2C in lung tumorigenesis identified in our previous work, we focused on other genes transcriptionally regulated by TFAP2C [7, 8]. Microarray data were analyzed for TFAP2C-affected genes in NSCLC cells by using the Gene Expression Omnibus database (GEO Series accession number GSE79228). As shown in Fig. 1, among the many genes upregulated or downregulated by TFAP2C knockdown (KD-TFAP2C, > 1.5-fold), we were interested in potential tumor suppressor genes. Of the 1152 genes upregulated by KD-TFAP2C, we excluded pseudogenes and nonfunctional genes. Of the 58 genes screened by analysis of gene ontology functional annotation based on hallmarks of cancer including apoptosis, anti-proliferation, and cell death, nine genes (GADD45B, PMAIP1, XAF1, CYR61, IL24, ATF3, DLC1, RHOB, and TNFAIP3) have been reported to act as tumor suppressive genes in several cancer types (Table 2).

**Table 2:** Tumor suppressive genes negatively regulated by TFAP2C in NSCLC

Gene	Location	Mechanism of tumorigenesis	Biological activity	Reference (PMID)
*GADD45B*	Chromosome 19, NC_000019.10 (2476125..2478259)	Activation of MTK1-p38 pathway	Cell cycle arrest and apoptosis	12933797
		Activation of CDKN1A expression	Cell cycle arrest and apoptosis	27572311
		Decrease of JNK and STAT5	Apoptosis and anti-proliferation	30279966
*PMAIP1*	Chromosome 18, NC_000018.10 (59899960..59904306)	Decrease of USP9X-MCL1 pathway	Apoptosis	24991768
		Activation of Beclin-1 via decrease of MCL1	Autophagy and cell death	21353614
		Activation of the mitochondrial apoptotic pathway via interaction with BIM	Apoptosis	26497683
*XAF1*	Chromosome 17, NC_000017.11 (6755408..6775647)	Activation of Beclin-1 and decrease of Akt pathway	Apoptosis, autophagy, and cell death	21788101
		Decrease of VEGF	Apoptosis, anti-proliferation, and anti-angiogenesis	24980821
*IL24*	Chromosome 1, NC_000001.11 (206897404..206904139)	Activation of eIF2α phosphorylation	Apoptosis	28461326
		Decrease of Wnt/β-catenin signaling	Apoptosis and anti-angiogenesis	23720015
*ATF3*	Chromosome 1, NC_000001.11 (212565334..212620777)	Activation of PMAIP1	Apoptosis	29352505
		Activation of Smad signaling	Cell death	20930144
*CYR61*	Chromosome 1, NC_000001.11 (85580761..85583967)	Activation of integrin α6-ROS-p38 pathway via activation of p53	Anti-proliferation	26028023
		Decrease of MMP-2	Anti-cell motility and anti-invasion	19632997
*DLC1*	Chromosome 8, NC_000008.11 (13083361..13604616, complement)	Decrease of RhoA	Anti-angiogenesis	28408355
		Decrease of VEGF via EGFR-MEK-HIF pathway	Anti-angiogenesis	20861185
*TNFAIP3*	Chromosome 6, NC_000006.12 (137866317..137883314)	Decrease of AKT1/TRIO/RAC1 pathway	Inhibition of EMT, anti-migration and anti-invasion	27676292
		Decrease of Wnt signaling via interaction with β-catenin destruction complex	Apoptosis and anti-angiogenesis	23671587
*RHOB*	Chromosome 2, NC_000002.12 (20447071..20449445)	Activation of E-cadherin and decrease of Vimentin	Anti-migration and anti-invasion	28253718
		Decrease of NF-κB signaling	Anti-angiogenesis, anti-migration and anti-invasion	20383180

### Verification of downregulation of selected tumor suppressor genes in NSCLC

**Fig. 2:**
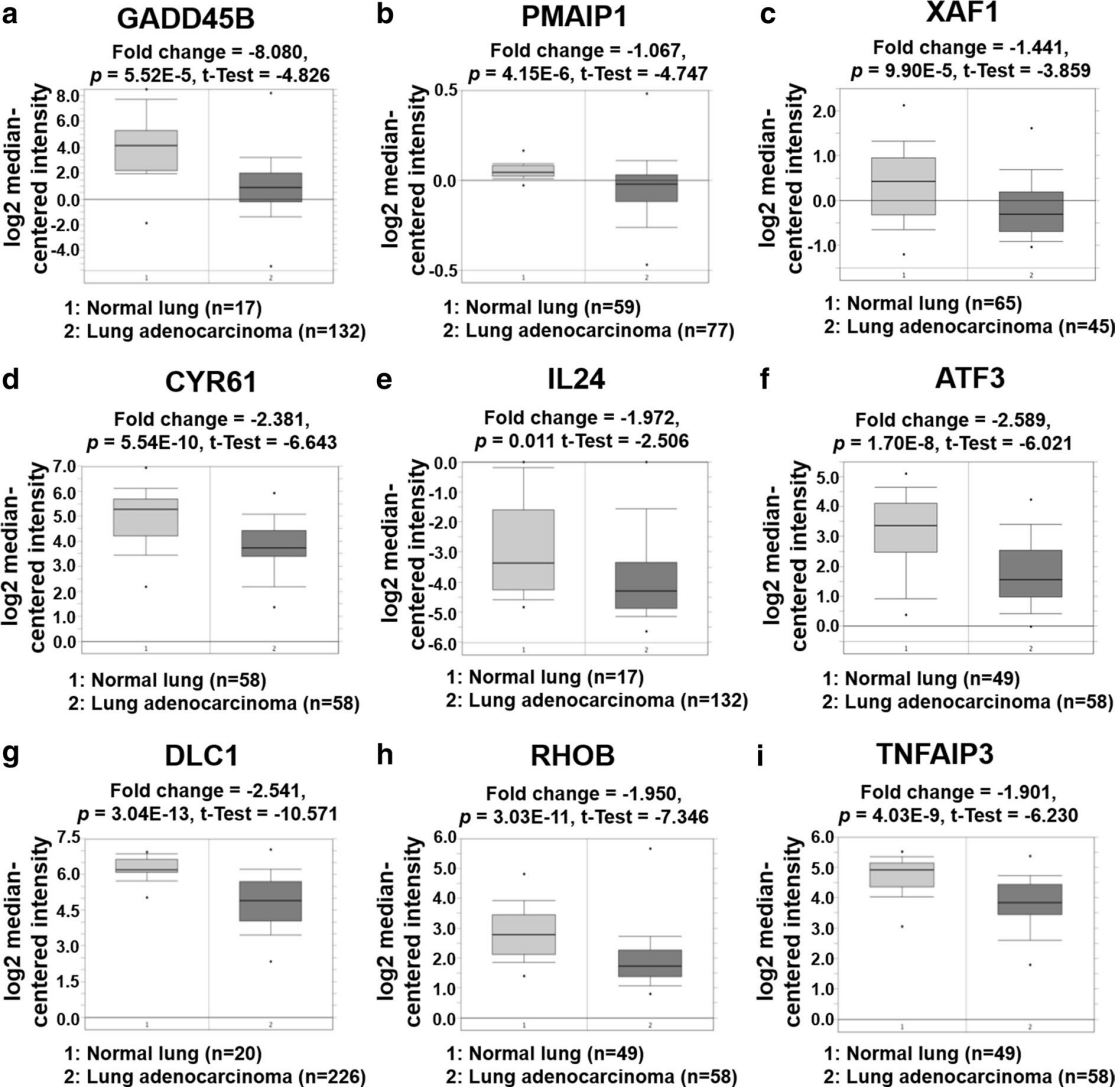
The expression patterns of nine selected genes in NSCLC based on analysis of the Oncomine database. **a** The Bhattacharjee dataset showed GADD45B downregulation in lung adenocarcinoma compared with the normal lung counterparts (fold change = –8.080, *p* = 5.52E−5). **b** The Weiss dataset showed PMAIP1 downregulation in lung adenocarcinoma (fold change = − 1.067, *p* = 4.15E−6). **c** The Hou dataset showed XAF1 downregulation in lung adenocarcinoma (fold change = − 1.441, *p* = 9.90E−5). **d** The Selamat dataset showed CYR61 downregulation in lung adenocarcinoma (fold change = − 2.381, *p* = 5.54E−10. **e** The Bhattacharjee dataset showed IL24 downregulation in lung adenocarcinoma (fold change = − 1.972, *p* = 0.011). **f** The Landi dataset showed ATF3 downregulation in lung adenocarcinoma (fold change = − 2.589, *p* = 1.70E−8). **g** The Okayama dataset showed DLC1 downregulation in lung adenocarcinoma (fold change = − 2.541, *p* = 3.04E−13). **h** The Landi dataset showed RHOB downregulation in lung adenocarcinoma (fold change = − 1.950, *p* = 3.03E−11). **i** The Selamat dataset showed TNFAIP3 downregulation in lung adenocarcinoma (fold change = − 1.901, *p* = 4.03E−9)

Next, to verify our results for the screening of nine genes as tumor suppressors for NSCLC, the genes were further investigated using Oncomine (http://www.oncomine.org), an online database for expression analysis of lung cancer tissues [18]. The database results indicated that GADD45B was downregulated by − 8.080-fold in lung adenocarcinomas [19], PMAIP1 was downregulated by − 1.067-fold in lung adenocarcinomas [20], XAF1 was downregulated by − 1.441-fold in large cell lung carcinomas [21], CYR61 was downregulated by − 2.381-fold in lung adenocarcinomas [22], IL24 was downregulated by − 1.972-fold in lung adenocarcinomas [19], ATF3 was downregulated by − 2.589-fold in lung adenocarcinomas [23], DLC1 was downregulated by − 2.541-fold in lung adenocarcinomas [24], RHOB was downregulated by − 1.950-fold in lung adenocarcinomas [23], and TNFAIP3 was downregulated by − 1.901-fold in lung adenocarcinomas [22], compared to normal lung counterparts, respectively (each *p* < 0.05, Fig. 2). These results showed that the nine genes were expressed at low levels in NSCLC tissues, indicating that they might provide clues for using the nine genes as potential prognostic markers for NSCLC tumor suppressors. Based on the results that GADD45B expression level was the lowest (− 8.080-fold change) in Oncomine data and PMAIP1 expression was highest (1.741-fold, data not shown) in KD-TFAP2C mediated microarray data, we selected GADD45B and PMAIP1 to represent the nine candidate genes in further study.

### Downregulation of GADD45B and PMAIP1 expression by TFAP2C in NSCLC cells

**Fig. 3:**
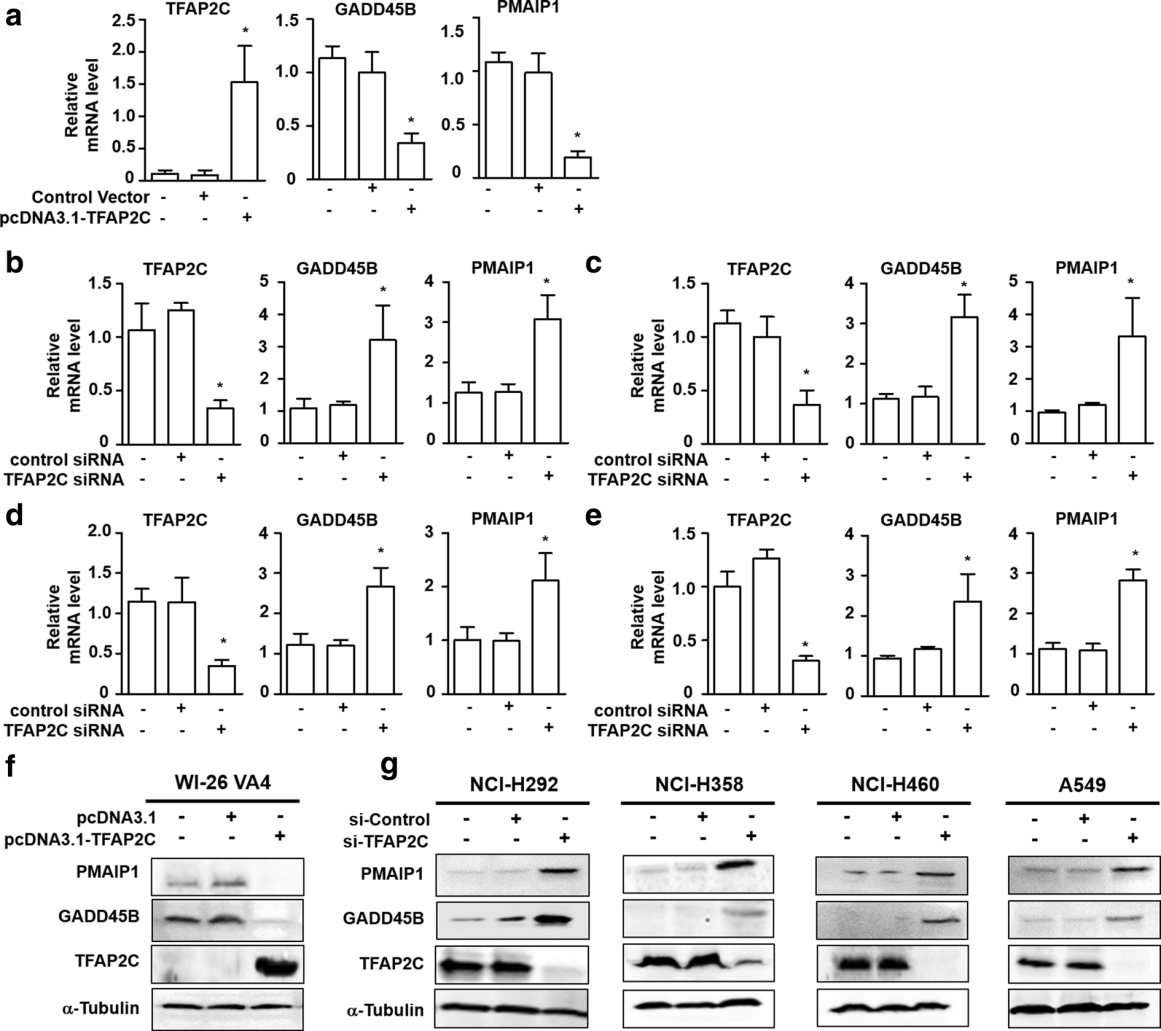
TFAP2C induces GADD45B and PMAIP1 downregulation in NSCLC cells. **a** The mRNA expression levels of GADD45B and PMAIP1 in TFAP2C-overexpressing WI-26 VA4 cells were examined by qRT-PCR. *p < 0.05 compared to non-treated cells or cells treated with control vector. **b**–**e** The mRNA expression levels of GADD45B and PMAIP1 in TFAP2C siRNA-treated NCI-H292 (**b**), NCI-H358 (**c**), NCI-H460 (**d**), and A549 (**e**) cells were examined by qRT-PCR. *p < 0.05 compared to non-treated cells or cells treated with control siRNA. **f** The protein expression levels of GADD45B and PMAIP1 in TFAP2C-overexpressing WI-26 VA4 cells were examined by Western blotting. **g** The protein expression levels of GADD45B and PMAIP1 in TFAP2C siRNA-treated NCI-H292, NCI-H358, NCI-H460, and A549 cells were examined by Western blotting

TFAP2C is primarily upregulated in NSCLC cells and tissues but downregulated in normal lung counterparts [7]. To confirm the microarray results, we analyzed GADD45B and PMAIP1 levels in TFAP2C-overexpressing normal lung cells (WI-26 VA4 cells) and TFAP2C siRNA-treated NSCLC cells (NCI-H292, NCI-H358, NCI-H460, and A549 cells) by using real time quantitative reverse transcription PCR (qRT-PCR). The mRNA levels of GADD45B and PMAIP1 were significantly decreased by TFAP2C overexpression in WI-26 VA4 cells (Fig. 3a) but were increased by treatment of TFAP2C siRNA in the four human NSCLC cell lines (Fig. 3b–e). Similarly, protein levels of GADD45B and PMAIP1 were downregulated in TFAP2C-overexpressing WI-26 VA4 cells (Fig. 3f) but were upregulated in TFAP2C siRNA-transfected NSCLC cells (Fig. 3g). These results suggest that GADD45B and PMAIP1 expressions were inhibited by TFAP2C and negatively correlated with the expression of TFAP2C.

### Increased cell proliferation in NSCLC cells by downregulation of GADD45B and PMAIP1 expression

**Fig. 4:**
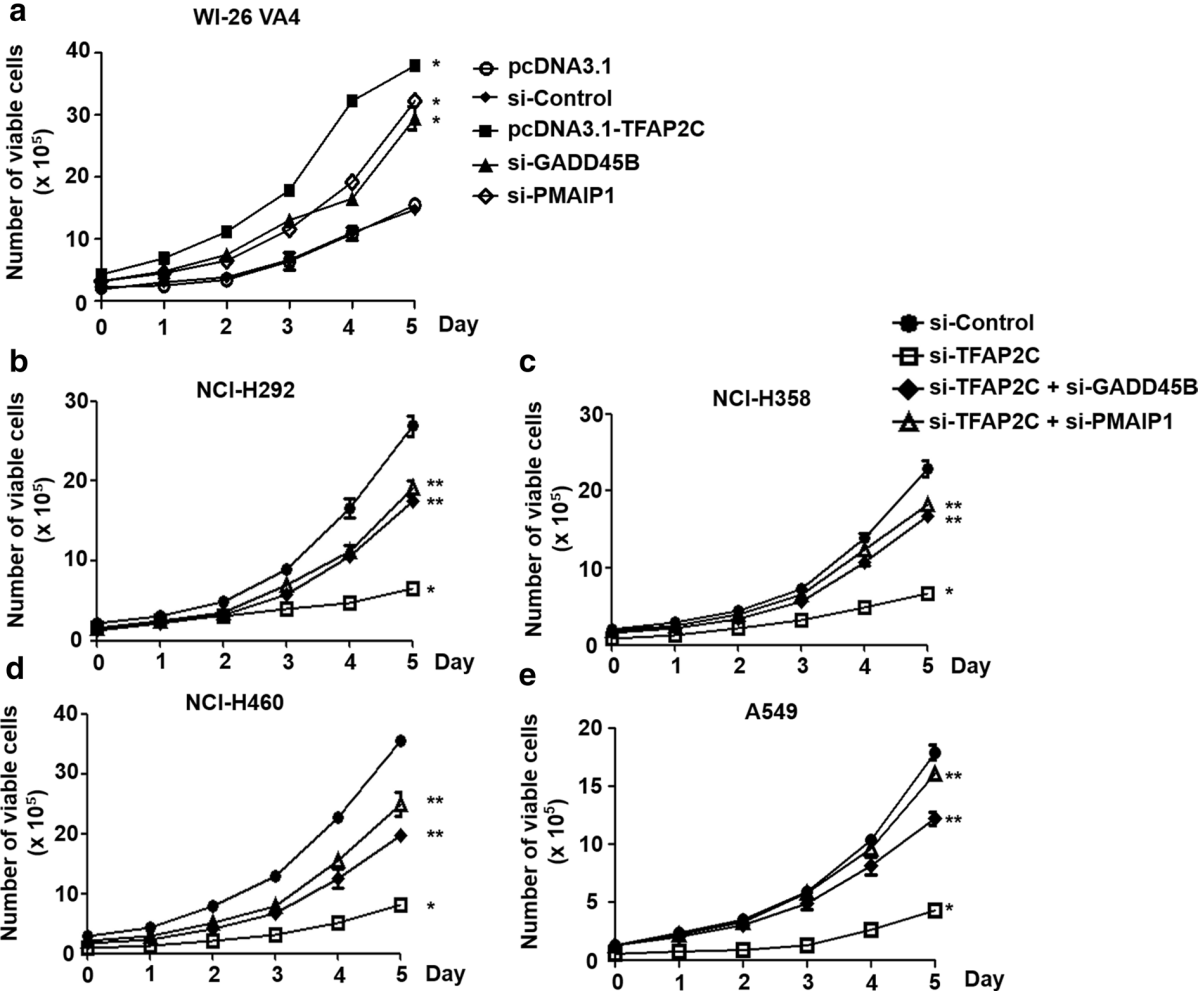
Short-term effects of TFAP2C, GADD45B, and PMAIP1 expression levels on NSCLC cell proliferation. **a** The effects of TFAP2C overexpression, GADD45B knockdown, or PMAIP1 knockdown on cell proliferation of WI-26 VA4 cells were measured by using cell viability assays. *p < 0.05 compared to cells treated with control vector or control siRNA. **b**–**e** The effects of TFAP2C, GADD45B, and/or PMAIP1 knockdown on cell proliferation of NCI-H292 (**b**), NCI-H358 (**c**), NCI-H460 (**d**), or A549 (**e**) cells were measured by using cell viability assays. *p < 0.05 compared to cells treated with control siRNA, **p < 0.05 compared to cells treated with TFAP2C siRNA

**Fig. 5:**
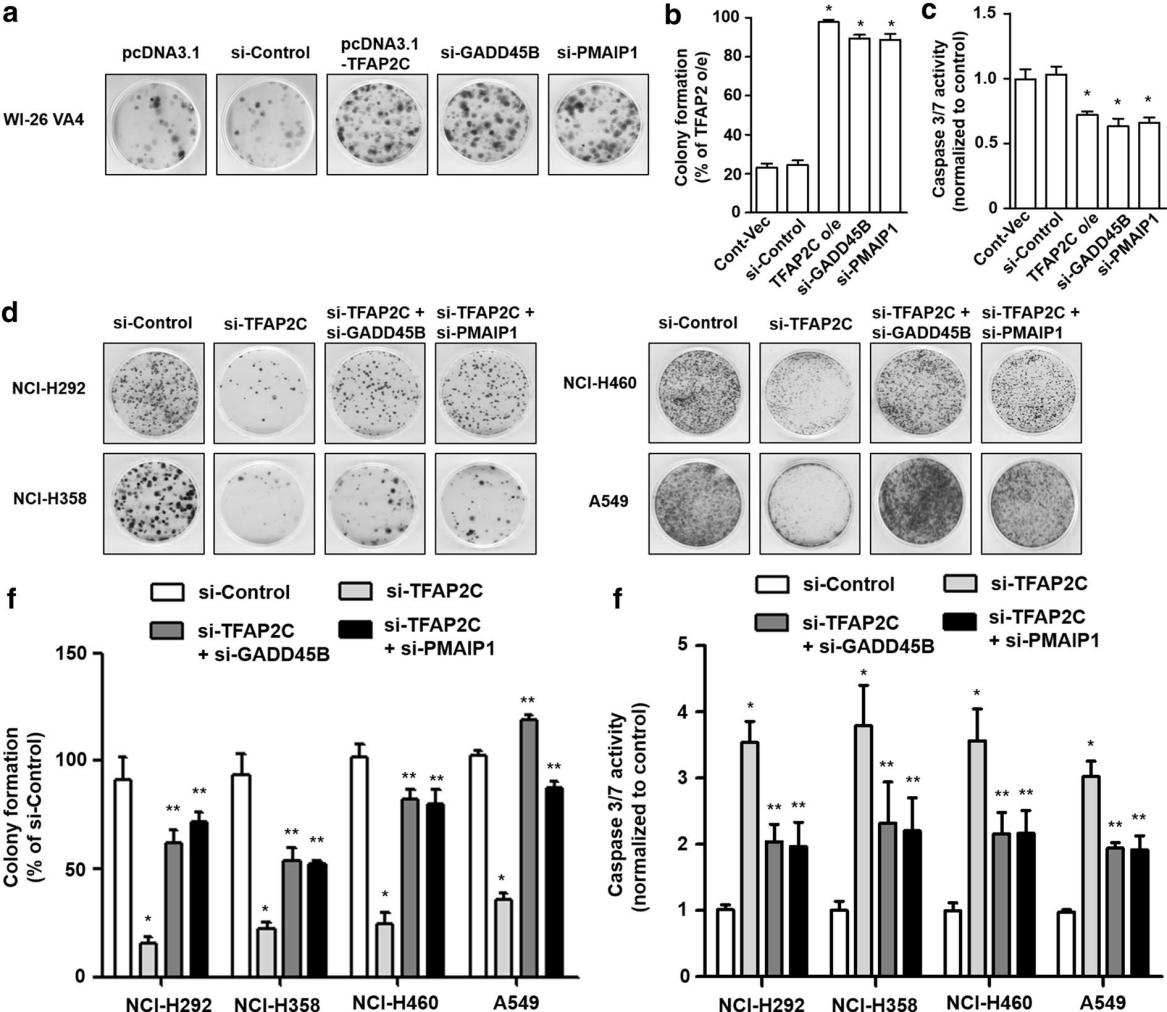
Long-term effects of TFAP2C, GADD45B, and PMAIP1 expression levels on NSCLC cell proliferation. **a** The effects of TFAP2C overexpression, GADD45B knockdown, or PMAIP1 knockdown on cell proliferation of WI-26 VA4 cells were measured using colony-forming assays. **b** Quantitative analysis of the number of WI-26 VA4 cell clones after TFAP2C overexpression, GADD45B knockdown, or PMAIP1 knockdown was performed. The colonies of TFAP2C-overexpressing cells set at 100%. *p < 0.05 compared to cells treated with control vector or control siRNA. **c** The effects of TFAP2C overexpression, GADD45B knockdown, or PMAIP1 knockdown on WI-26 VA4 cell apoptosis were determined using caspase 3/7 assays. *p < 0.05 compared to cells treated with control vector or control siRNA. **d** The effects of TFAP2C, GADD45B, and/or PMAIP1 knockdown on cell proliferation of NCI-H292, NCI-H358, NCI-H460, or A549 cells were measured by using colony-forming assays. Representative images of each group are presented. **e** Quantitative analysis of the number of four NSCLC cell clones after the treatments was performed. The colonies of non-treated control cells set at 100%. *p < 0.05 compared to cells treated with control siRNA, **p < 0.05 compared to cells treated with TFAP2C siRNA. **f** The effects of TFAP2C, GADD45B, and/or PMAIP1 knockdown on apoptosis of NCI-H292, NCI-H358, NCI-H460, or A549 cells were measured by using caspase 3/7 assays. *p < 0.05 compared to cells treated with control siRNA, **p < 0.05 compared to cells treated with TFAP2C siRNA

It has been reported that GADD45B and PMAIP1 have significant roles in anti-proliferation and apoptosis, suggesting that they may function as tumor suppressors [25, 26]. Based on their inhibitory effects on cell proliferation, we analyzed the short-term effects of GADD45B and PMAIP1 overexpressions or knockdowns on cell growth of normal lung cells and the four human NSCLC cell lines using cell viability assays. It was observed that the number of viable cells was increased by TFAP2C-overexpressing WI-26 VA4 cells compared to that in control cells at day 5, and those of the viable cells in GADD45B or PMAIP1 siRNA-treated cells were considerably increased, nearly up to the level in the TFAP2C-overexpressing cells (Fig. 4a). In contrast, the number of survived cells in TFAP2C siRNA-treated NSCLC cells was lower, compared to control siRNA-treated cells, at day 5, and that of the survived cells was significantly rescued by further treatment of GADD45B or PMAIP1 siRNA (Fig. 4b–e). In addition, colony-forming assays were performed to determine whether the expressions of GADD45B and PMAIP1 regulated by TFAP2C were associated with long-term effects on cell proliferation. We found that the colony-forming capacity in WI-26 VA4 cells was enhanced by TFAP2C overexpression and GADD45B or PMAIP1 inhibition compared to that in control cells (Fig. 5a, b). In addition, we determined whether the expressions of TFAP2C, GADD45B, and PMAIP1 were associated with the apoptosis by examining the activation of caspase 3/7. The activities of caspase 3/7 in WI-26 VA4 cells were decreased by TFAP2C overexpression and GADD34B or PMAIP1 inhibition compared to control cells (Fig. 5c). Conversely, the colony formation ability in NCI-H292, NCI-H358, NCI-H460, and A549 cells was diminished, compared to that in control siRNA-treated cells, by treatment of TFAP2C siRNA, whereas colony formation was partially, but significantly, recovered by further GADD45B or PMAIP1 siRNA treatment (Fig. 5d, e). Moreover, the activities of caspase 3/7 in NSCLC cell lines were increased by TFAP2C siRNA treatment, compared to cells treated with control siRNA (Fig. 5f). The increased activities of caspase 3/7 by TFAP2C knockdown were reduced by further treatment of GADD45B or PMAIP1 siRNA. However, we observed that inhibition of GADD45B or PMAIP1 alone did not affect colony formation ability in four NSCLC cell lines compared to that in control cells (Additional file 1: Fig. S1). These results indicate that GADD45B and PMAIP1 expressions have important roles in NSCLC cell proliferation.

### Increased cell motility in NSCLC cells by downregulation of GADD45B and PMAIP1 expression

**Fig. 6:**
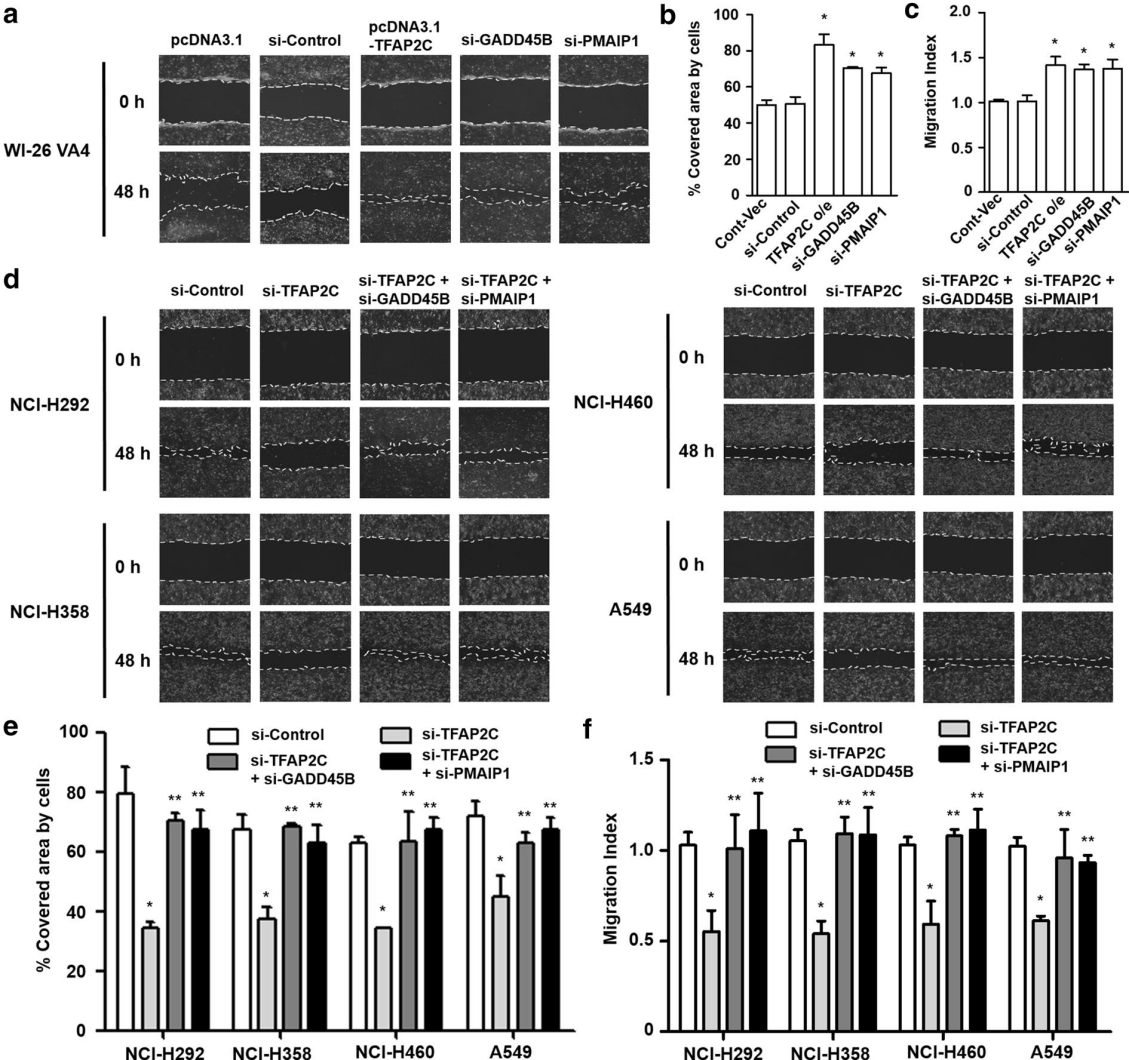
The effects of TFAP2C, GADD45B, and PMAIP1 expression levels on NSCLC cell motility. **a** The effects of TFAP2C overexpression, GADD45B knockdown, or PMAIP1 knockdown on cell motility of WI-26 VA4 cells were measured by using wound-healing assays. Representative images of each group are presented. **b** The graphs show the percentage of the available area covered by WI-26 VA4 cells from five randomly selected images. Representative images of each group at different times are presented. *p < 0.05 compared to cells treated with control vector or control siRNA. **c** The effects of TFAP2C overexpression, GADD45B knockdown, or PMAIP1 knockdown on cell migration capacity of WI-26 VA4 cells were measured by using transwell cell migration assays. *p < 0.05 compared to cells treated with control vector or control siRNA. **d** The effects of TFAP2C, GADD45B, and/or PMAIP1 knockdown on cell motility of NCI-H292, NCI-H358, NCI-H460, or A549 cells were measured by using wound-healing assays. Representative images of each group are presented. **e** The graphs show the percentage of the available area covered by four NSCLC cell lines from five randomly selected images. *p < 0.05 compared to cells treated with control siRNA, **p < 0.05 compared to cells treated with TFAP2C siRNA. **f** The effects of TFAP2C, GADD45B, and/or PMAIP1 knockdown on cell migration capacity of NCI-H292, NCI-H358, NCI-H460, or A549 cells were measured by using transwell cell migration assays. *p < 0.05 compared to cells treated with control siRNA, **p < 0.05 compared to cells treated with TFAP2C siRNA

Cell motility, which is highly correlated with epithelial-mesenchymal transition (EMT) invasion, and migration, is one of the common features related to cancer aggressiveness and malignant progression [27]. To determine the effects of GADD45B and PMAIP1 expression levels on cell motility, we conducted wound-healing assays and transwell cell migration assays in normal lung cells and NSCLC cells. We observed that WI-26 VA4 cells treated with TFAP2C overexpression, GADD45B siRNA, or PMAIP1 siRNA showed enhanced cell motility based on the time-dependent ratio of scratch coverage and migration capacity compared to those for control cells (Fig. 6a–c). In contrast, compared to those for control siRNA-treated cells, the covered areas and migrated cells for each of the four human NSCLC cell lines decreased with treatment of TFAP2C siRNA, and the uncovered areas were significantly filled and cell migration capacity was recovered after further treatment with GADD45B or PMAIP1 siRNA (Fig. 6d–f). The area-coverage tendencies, by cell line and based on repeated experiments, are depicted. However, we observed that inhibition of GADD45B or PMAIP1 alone did not affect wound-healing ability in four NSCLC cell lines compared to that in the control cells (Additional file 2: Fig. S2). Overall, these results indicate that the TFAP2C-suppressed expressions of GADD45B and PMAIP1 are positively involved in NSCLC malignancy including EMT, invasion, and migration.

## Discussion

In the current study, we investigated the involvements of TFAP2C and TFAP2C-regulated genes in lung tumorigenesis. Through an analysis of microarray data, we identified nine tumor suppressors, GADD45B, PMAIP1, XAF1, CYR61, IL24, ATF3, DLC1, RHOB and TNFAIP3, that were downregulated by TFAP2C overexpression in NSCLC cells. The low expression levels of the nine genes in lung cancer were confirmed by examination of the Oncomine data. Although all nine genes were involved in cancer cell death, tumor growth, angiogenesis, and EMT through various mechanisms [26, 28–36], we focused on GADD45B and PMAIP1 (also known as Noxa) as representative tumor suppressors in NSCLC tumorigenesis. Our results show that knockdowns of GADD45B and PMAIP1 conferred cell proliferation, colony-forming capacity, and cell motility on normal lung cells and NSCLC cells. Taken together, the results suggest that GADD45B and PMAIP1, when negatively regulated by TFAP2C, can act as tumor suppressive factors inhibiting NSCLC tumorigenesis.

GADD45B functions as a DNA damage sensor and has been studied as a positive regulator of apoptosis under various stimuli. Although GADD45B might be up-regulated and responsible for carcinogenesis under DNA damage response in a few cases [37, 38], many studies have presented that downregulation of GADD45B contributes to tumorigenesis and malignant development in human cancers. Another study reported that a loss of GADD45B was associated with upregulation of JNK and signal transducer and activator of transcription (STAT) 5, resulting in cell proliferation and inhibition of apoptosis in tumorigenesis of chronic myeloid leukemia [25]. In a study of functional analyses of chemotherapeutic agents, GADD45B was positively involved in G2/M-phase arrest and apoptosis through induction of cyclin-dependent kinase inhibitor 1A expression [39]. Overexpression of GADD45B has enhanced Fas-induced apoptosis through increased interaction of p38 with Rb protein [40]. In addition, the expression of GADD45B can be upregulated by Smad2, Smad3, and Smad4 signaling pathways stimulated by the transforming growth factor beta (TGF-β) signaling [41]. Another study demonstrated that STAT3 could bind to upstream regulatory elements of the GADD45B gene to function as a transcriptional repressor of GADD45B [42]. A recent study reported that the mRNA of STAT3 is targeted by miR-519a [43]. Interestingly, based our microarray dataset, we observed that miR-519a was slightly upregulated by TFAP2C knockdown. These clues suggest that low expression levels of GADD45B might be indirectly due to TFAP2C-mediated downregulation of miR-519a, although we could not exclude the possibility that TFAP2C can act as a direct repressor of GADD45B.

PMAIP1 belongs to the BH3-only pro-apoptotic protein of the Bcl-2 family and upregulation of PMAIP1 leads to apoptosis in a variety of cancers. Downregulation of PMAIP1 has been shown to promote the expression of ubiquitin-specific peptidase 9, X-linked, and myeloid cell leukemia 1, resulting in apoptosis in NSCLC cells [26]. In addition, it has been reported that PMAIP1 has an important role in the induction of apoptosis and autophagy, and was shown to be downregulated in adenoid cystic carcinoma [44]. Another study demonstrated that PMAIP1 mRNA can be targeted by miR-21 in gastric carcinoma, resulting in the inhibition of proliferation, invasion, and migration [45]. In particular, a study using chromatin immunoprecipitation showed that activating transcription factor (ATF) 3 and ATF4 could be putative transcription factors responsible for the expression of PMAIP1 [46]. That suggestion was supported by another study in which cisplatin-induced ATF3 in head and neck squamous cell carcinoma cells resulted in induction of apoptosis accompanied by an increased level of PMAIP1 through cooperative binding of ATF3 and ATF4 to the PMAIP1 promoter [47]. As shown in Figs. 1 and 2, we also found ATF3 to be upregulated by TFAP2C knockdown, based on the analysis of the TFAP2C-related microarray dataset. Although further molecular studies into the interplay between TFAP2C, ATF3, and PMAIP1 would be required, we suggest that oncogenic transcription factor TFAP2C might contribute to NSCLC tumorigenesis by downregulation of PMAIP1 in an ATF3-dependent manner.

## Conclusion

There have been many studies seeking to identify molecular targets useful in elucidating the molecular mechanisms underlying tumorigenesis and malignant development of NSCLC. The results of the present study have shown that TFAP2C can contribute to NSCLC tumorigenesis via downregulation of many tumor suppressors including GADD45B, PMAIP1, XAF1, CYR61, IL24, ATF3, DLC1, RHOB, and TNFAIP3. For the first time, we report that GADD45B and PMAIP1, which are suppressed by TFAP2C, could be highly responsible for cell proliferation and cell motility in NSCLC. We propose that GADD45B and PMAIP1 be considered putative tumor suppressive factors in NSCLC that might be useful as prognostic markers during NSCLC therapy.

## Additional files

**Additional file 1: Fig. S1.** The effects of TFAP2C, GADD45B and PMAIP1 expression levels on NSCLC cell proliferation.

**Additional file 2: Fig. S2.** The effects of TFAP2C, GADD45B and PMAIP1 expression levels on NSCLC cell motility.

## Data Availability

All data or data analyzed during this study are included in this published article (and its additional information files).

## Abbreviations

NSCLC: non-small cell lung cancer
GADD45B: growth arrest and DNA-damage-inducible beta
PMAIP1: phorbol-12-myristate-13-acetate-induced protein 1
TFAP2C: transcription factor activating enhancer-binding protein 2C
RT: reverse transcription
SD: standard deviation
STAT: signal transducer and activator of transcription
ATF: activating transcription factor
