# Supplementary material for: TFAP2C increases cell proliferation by downregulating GADD45B and PMAIP1 in non-small cell lung cancer cells
Source: Biol Res. 2019 Jul 11;52:35. doi: 10.1186/s40659-019-0244-5 (PMC6625030; doi:10.1186/s40659-019-0244-5)
Supplement: Supplementary file 2 — Additional file 2: Fig. S2. The effects of TFAP2C, GADD45B and PMAIP1 expression levels on NSCLC cell motility. [file 40659_2019_244_MOESM2_ESM.docx]

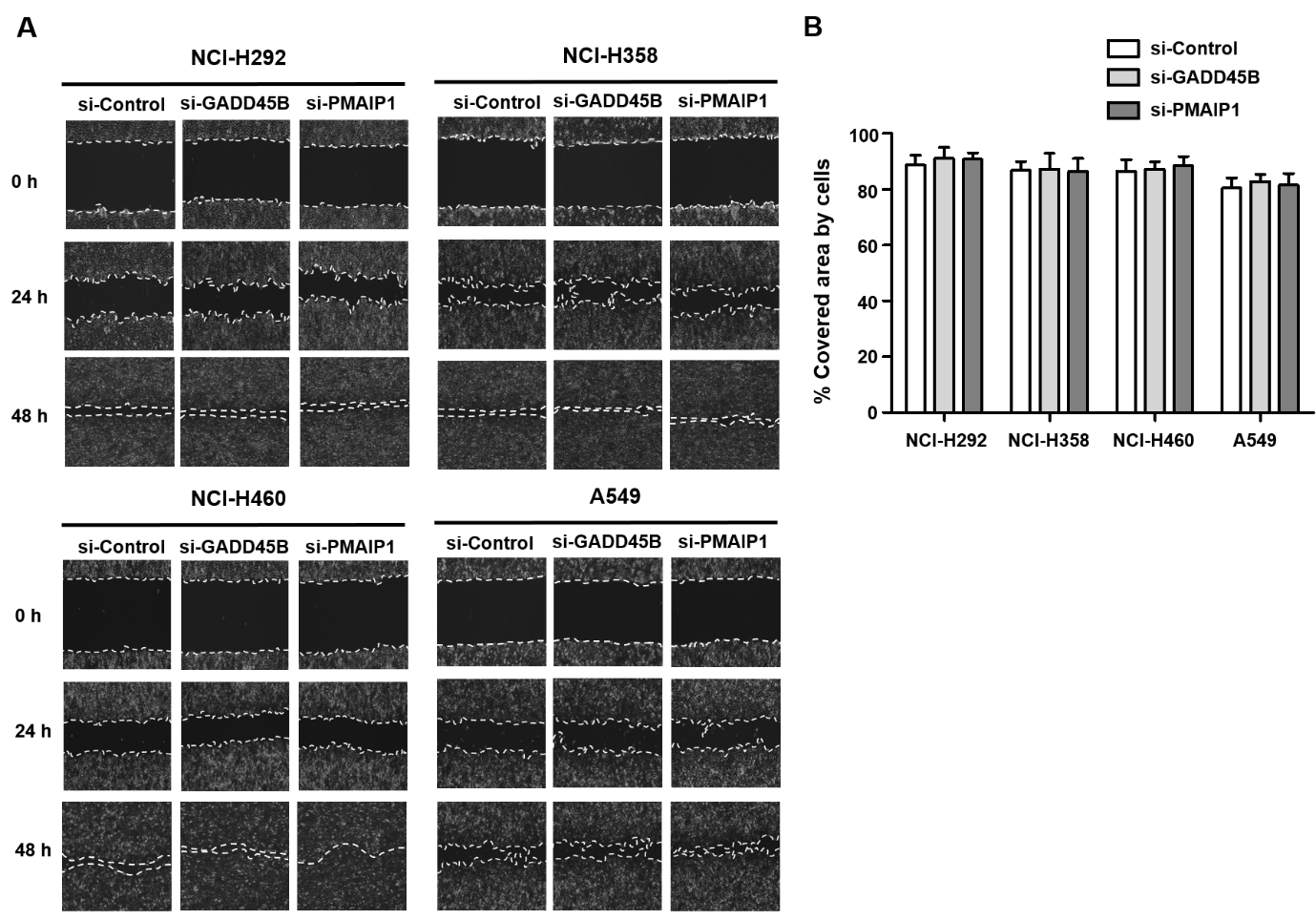


**Fig. S2.** The effects of TFAP2C, GADD45B and PMAIP1 expression levels on NSCLC cell motility. (A) The effects of GADD45B knockdown, or PMAIP1 knockdown on cell motility of NCI-H292, NCI-H358, NCI-H460, or A549 cells were measured by using wound-healing assays. Representative images of each group at different time points are presented. (B) The graphs show the percentage of the area covered by cells at a 48 h for NSCLC cells from five randomly selected images. Representative images of each group at different time points are presented.
